# Adjuvant tegafur-uracil (UFT) or S-1 monotherapy for advanced gastric cancer: a single center experience

**DOI:** 10.1186/s12957-021-02233-2

**Published:** 2021-04-17

**Authors:** Hung-Hsuan Yen, Chiung-Nien Chen, Chi-Chuan Yeh, I-Rue Lai

**Affiliations:** 1 Department of Surgery, National Taiwan University Biomedical Park Hospital, Hsinchu County, Taiwan; 2 Department of Surgery, National Taiwan University Hospital, No. 7 Chung-Shan South Rd, Zhongzheng District, Taipei, 10002 Taiwan; 3 Department of Medical Education, National Taiwan University Hospital, Taipei, Taiwan; 4 Graduate Institute of Anatomy and Cell Biology, College of Medicine, National Taiwan University, Taipei, Taiwan

**Keywords:** Adjuvant chemotherapy, Gastric cancer, S-1, Tegafur, UFT

## Abstract

**Background:**

Adjuvant tegafur-gimeracil-oteracil (S-1) is commonly used for gastric cancer in Asia, and tegafur-uracil (UFT) is another oral fluoropyrimidine when S-1 is unavailable. The real-world data of adjuvant UFT has less been investigated.

**Methods:**

Patients with pathological stage II-IIIB (except T1) gastric cancer receiving adjuvant UFT or S-1 monotherapy after D2 gastrectomy were included. Usage of UFT or S-1 was based on reimbursement policy of the Taiwanese healthcare system. The characteristics, chemotherapy completion rates, and 5-year recurrence-free survival (RFS) and overall survival (OS), were compared between these two groups.

**Results:**

From 2005 to 2016, 86 eligible patients were included. Most tumor characteristics were similar between the UFT group (*n* = 37; age 59.1 ± 13.9 years) and S-1 group (*n* = 49; age 56.3 ± 10.7 years), except there were significantly more Borrmann type III/IV (86.5% versus 67.3%; *p* = 0.047) and T4 (56.8% versus 10.2%; *p* < 0.001) lesions in the UFT group than in the S-1 group. The chemotherapy complete rates were similar in the two groups. The 5-year RFS was 56.1% in the UFT group and 59.6% in the S-1 group (*p* = 0.71), and the 5-year OS was 78.3% in the UFT group and 73.1% in the S-1 group (*p* = 0.48). The hazard ratio of adjuvant chemotherapy (S-1 versus UFT) on RFS was 1.25 (95% confidence interval = 0.53-2.94) when Borrmann type and T and N stages were adjusted.

**Conclusions:**

This small cohort study showed adjuvant UFT, and S-1 monotherapy had a comparable long-term outcome for pathological stage II-IIIB gastric cancer following D2 gastrectomy.

**Supplementary Information:**

The online version contains supplementary material available at 10.1186/s12957-021-02233-2.

## Background

Gastric cancer has been the 3rd most deadly cancer worldwide [1]. Radical gastrectomy with lymph node dissection is the treatment of choice for resectable gastric cancer [2], and adjuvant chemotherapy is recommended for pathological stage II-IIIB gastric cancer based on several randomized controlled trials (RCTs), Japanese gastric cancer treatment guidelines, and Korean practice guideline for gastric cancer [3–12].

Dihydropyrimidine dehydrogenase (DPD) inhibitory fluoropyrimidine (DIF), the oral form prodrug of 5-flurouracil (5-FU), has been developed since 1980 [10], and is currently the most important chemotherapeutic agent used in adjuvant chemotherapy for advanced gastric cancer. Tegafur-uracil (UFT) is the first generation of oral DPD DIF, followed by tegafur-gimeracil-oteracil (S-1), the next generation of oral DPD DIF. S-1 contains oteracil that inhibits phosphorylation of 5-FU within the gastrointestinal mucosal cells and thus theoretically reduces the gastrointestinal toxicity [13].

UFT was firstly used as an adjuvant monotherapy for pathological T2N1-2 gastric cancer in 1997 [9], but the introduction of S-1 has replaced UFT gradually since 2001. In addition, S1 monotherapy has further become the standard adjuvant therapy for advanced gastric cancer in 2007 since the results of Adjuvant Chemotherapy Trial of TS-1 for Gastric Cancer (ACTS-GC) was published [7, 8, 14]. In Taiwan, UFT was approved for advanced gastric cancer and reimbursed by the Taiwanese healthcare system in October 2000; on the other hand, S-1 was approved for advanced gastric cancer based on the same inclusion criteria in the ACTS-GC in April 2010 but was not reimbursed until December 2016. Although both UFT and S-1 are indicated for advanced gastric cancer as adjuvant monotherapy in Taiwan, real-world experiences and comparisons of efficacy and tolerance between these two oral DPD DIFs remain suboptimal and are only limited to sub-group analysis in one clinical trial, the Stomach cancer Adjuvant Multi-Institutional group Trial (SAMIT) [15].

In the era of coronavirus disease (COVID-19) pandemic, the supply chain of chemotherapeutic agents could be no longer guaranteed. Re-evaluation of the existing alternative in the adjuvant treatment may be necessary. In this study, we shared our experiences of using UFT or S-1 monotherapy as adjuvant treatment for pathological stage II-IIIB gastric cancer after D2 gastrectomy, based on the unique historical cohorts formed by the different timings when UFT and S-1 were available in Taiwan.

## Methods

### Study design and identification of the study cohort

**Fig. 1:**
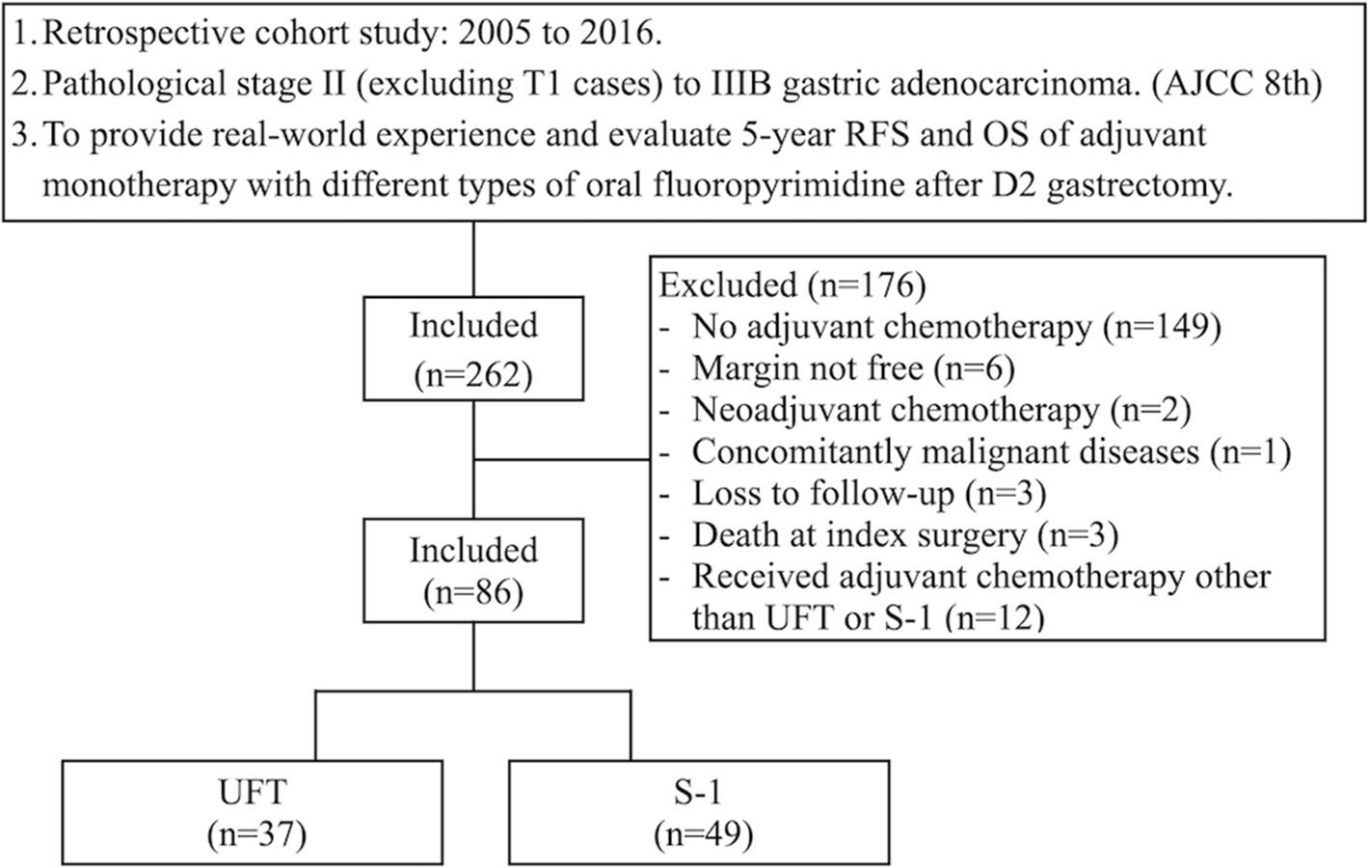
Flow diagram of the subjects for the study. AJCC, American Joint Cancer Committee; RFS, recurrence-free survival; OS, overall survival; UFT, tegafur-uracil; S-1, tegafur-gimeracil-oteracil

This is a retrospective cohort study for patients receiving adjuvant UFT or S-1 monotherapy after D2 gastrectomy by two senior surgeons (I.-R. L. and C.-N. C) in a tertiary referral medical center from 2005 to 2016. Patients with pathological stage II-IIIB gastric adenocarcinoma (excluding T1 cases), based on the American Joint Committee on Cancer (AJCC) 8th staging system, were included in the study. The surgical procedure followed the principles provided by Japanese Gastric Cancer Association [11, 16, 17]. The patients, who, declined adjuvant chemotherapy, had a positive margin on final pathology report, were treated with neoadjuvant chemotherapy, had concomitant malignancy, were lost to follow-up (no-show since the first postoperative outpatient follow-up), had operative mortality (death occurring < 1 month of the index gastrectomy), or received adjuvant chemotherapy other than UFT or S-1, were excluded from the analysis. For patients who were lost to follow-up or had operative mortality, discussion and decision of the adjuvant chemotherapy were not made. A total of 262 patients had been screened initially and 86 patients who met the criteria were included (Fig. 1). The study was approved by the Institutional Review Board of National Taiwan University Hospital.

### Treatment and follow-up

All the included patients were evaluated for and began adjuvant chemotherapy within 6 weeks after surgery. The eligible patients were classified into two groups, the UFT and S-1 groups, based on the type of adjuvant chemotherapy. During the period between 2005 and 2010, UFT was the only available oral fluoropyrimidine for advanced gastric cancer as adjuvant chemotherapy. Then S-1, the next generation of oral fluoropyrimidine, was introduced to our hospital since 2010. The UFT group was given oral UFT 267 mg/m^2^ in two or three doses per day for 28 days a course for 24 course (96 weeks) [15], and the S-1 group was given oral S-1 80 mg/m^2^ in two doses per day for 28 days followed by 14 days rest for 8 courses (48 weeks) [7, 8]. The dosage of UFT or S-1 was reduced if any intolerable adverse event or ≥ grade 3 adverse event occurred. Although we used the same ACTS-GC protocol in the S-1 group, we always extended the total number of chemotherapy course from 8 courses (48 weeks) to 12 courses (72 weeks) if dose de-escalation was required [18, 19]. The general principles for discontinuing UFT or S-1 in our routine practice included the following: (1) recurrence or death; (2) adverse events, more than 29 days of unresolved events that prevented starting or continuing a course; and (3) patients’ preference. Completion of the adjuvant chemotherapy was defined as patients who had finished 24 courses for UFT or 8 courses for S-1 regardless of any dose reduction or schedule modification.

All patients received outpatient follow-up every 3 months during the first 2 years and then every 6 months from the third to fifth year postoperatively. Weight recording, symptom inquiry, and physical examination, were conducted at every outpatient follow-up. Blood sample for routine complete blood count, basic chemistry panel (liver and renal functions), and tumor marker (carcinoembryonic antigen) was collected every time before follow-up. Esophagogastroduodenoscopy was routinely performed at 1 year after surgery, and computed tomography scan of the abdomen and pelvis was performed every 6 months until the fifth year after surgery. The schedule of computed tomography scan, esophagogastroduodenoscopy or outpatient visit would be brought forward or increased in frequency if patients exhibited signs suspicious of recurrence. All these follow-up data were kept in either paper-based or electronic medical record.

The recurrence-free survival (RFS) was defined as the time between chemotherapy start date and the first event (all-cause death, recurrence of gastric cancer, or occurrence of a second cancer), and the overall survival (OS) was defined as the time between chemotherapy start date and all-cause death. The long-term follow-up data were acquired from the electronic medical record and database maintained by Cancer Registry, Cancer Administration and Coordination Center in our hospital. Patients lost to follow-up were censored. All the adverse events were reported based on the Common Terminology Criteria for Adverse Events, version 4.03.

### Statistical analysis

Basic characteristics, including demographic data, operative methods, tumor classifications [20], pathological features and staging [16], and follow-up duration, were recorded and compared between the UFT and S-1 groups. The adjuvant chemotherapy completion rates, sites of the first recurrence, and adverse events were also compared between these two groups. The chi-squared test or Fisher’s exact test was used for qualitative variables as appropriate, and the Student’s *t* test was used for quantitative variables. We assessed time-to-event endpoint with the Kaplan-Meier method, and estimated the hazard ratio (HR) and its two-sided 95% confidence interval (CI) with the Cox regression model. Multivariate analysis for RFS was performed using Cox proportional hazards model; risk factors significant (*p* < 0.05) in the univariate analysis (Borrmann type) or considered important (type of chemotherapy, T, and N stages) were included. All statistical tests were two-sided, and a *p* value < 0.05 denoted statistical significance. All statistical analyses were performed using R version 3.4.0.

## Results

From 2005 to 2016, totally 86 gastric cancer patients with pathological stage II-IIIB (AJCC 8th edition) receiving adjuvant UFT or S-1 monotherapy after D2 gastrectomy by two senior surgeons (I.-R. L. and C.-N. C) were included in the study (Fig. 1). The UFT and S-1 groups comprised 37 and 49 patients, respectively, and there was no crossover between these two groups (Fig. 1).

Table 1 summarizes the basic characteristics of the UFT and S-1 groups. There was no statistical difference in most variables, including sex ratio, age, operative method, and most tumor characteristics, except for the Bormann type, T stage, and follow-up duration. More Borrmann type III/IV (86.5% versus 67.3%; *p* = 0.047) and T4 lesions (56.8% versus 10.2%; *p* < 0.001) were observed in the UFT group than in the S-1 group. The follow-up duration was longer in the UFT group (65.9 ± 36.9 months) than in the S-1 group (43.3 ± 22.6 months; *p* = 0.001). There were more pathological stage III gastric cancers in the UFT group than in the S-1 group (70.3% versus 55.1%) but with no statistical significance (*p* = 0.23).

**Table 1:** Basic characteristics between patients receiving UFT or S-1 as adjuvant chemotherapy

	All	(*n* = 86)	UFT	(*n* = 37)	S-1	(*n* = 49)	*p* value
Sex (male)	56	(65.1)	25	(67.6)	31	(63.3)	0.85
Age (years old)	57.5	± 12.2	59.1	± 13.9	56.3	± 10.7	0.31
Operative method							0.10
Distal gastrectomy	65	(75.6)	26	(70.3)	39	(79.6)	
Total gastrectomy	18	(20.9)	11	(29.7)	7	(14.3)	
Proximal gastrectomy	3	(3.5)	0	(0)	3	(6.1)	
Site							0.33
Antrum and low body	61	(70.9)	26	(70.3)	35	(71.4)	
Middle and high body	18	(20.9)	9	(24.3)	9	(18.4)	
Whole stomach	1	(1.2)	1	(2.7)	0	(0)	
Remnant	6	(7.0)	1	(2.7)	5	(10.2)	
Size (cm)	4.4	± 2.0	4.3	± 2.0	4.6	± 2.1	0.49
Bormann type							0.047*
I or II	21	(24.4)	5	(13.5)	16	(32.7)	
III or IV	65	(75.6)	32	(86.5)	33	(67.3)	
Lauren classification							0.49
Intestinal type	31	(36.0)	13	(35.1)	18	(36.7)	
Diffuse type	39	(45.3)	19	(51.4)	20	(40.8)	
Mixed type	16	(18.6)	5	(13.5)	11	(22.4)	
Cell differentiation							0.54
Well-differentiated	7	(8.2)	4	(11.1)	3	(6.1)	
Moderately differentiated	23	(27.1)	11	(30.6)	12	(24.5)	
Poorly- or un-differentiated	55	(64.7)	21	(58.3)	34	(69.4)	
LN metastasis (number)	4.5	± 5.7	5.2	± 7.8	3.9	± 3.3	0.35
LN harvested (number)	34.9	± 18.6	33.9	± 19.3	35.6	± 18.2	0.67
Lymphovascular invasion	56	(65.1)	25	(67.6)	31	(63.3)	0.85
Perineural invasion	57	(66.3)	25	(67.6)	32	(65.3)	0.99
T stage (AJCC 8^th^)							<0.001*
1	0	(0)	0	(0)	0	(0)	
2	19	(21.8)	8	(21.6)	11	(22.4)	
3	42	(48.3)	8	(21.6)	33	(67.3)	
4	26	(29.9)	21	(56.8)	5	(10.2)	
N stage (AJCC 8^th^)							0.48
0	2	(2.3)	0	(0)	2	(4.1)	
1	33	(38.4)	15	(40.5)	18	(36.7)	
2	36	(41.9)	14	(37.8)	22	(44.9)	
3	15	(17.4)	8	(21.6)	7	(14.3)	
Pathological stage (AJCC 8^th^)							0.23
II	33	(38.4)	11	(29.7)	22	(44.9)	
III	53	(61.6)	26	(70.3)	27	(55.1)	
Follow-up (months)	52.6	± 31.2	65.9	± 36.9	43.3	± 22.6	0.003*

Data are mean ± SD or *n* (%)

*UFT* tegafur-uracil, *S-1* tegafur-gimeracil-oteracil, *LN* lymph node, *AJCC* American Joint Cancer Committee

*p value < 0.05 after comparing between the UFT and S-1 groups

**Fig. 2:**
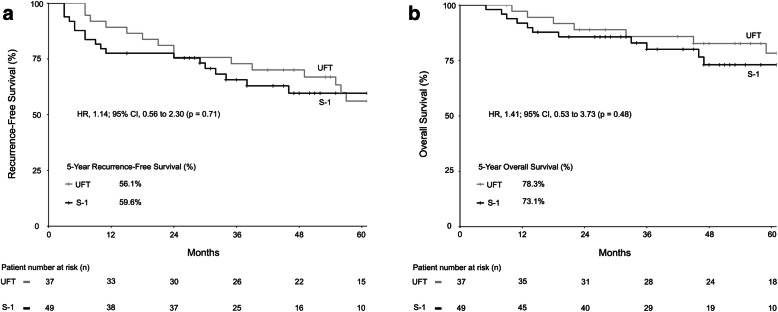
Kaplan–Meier analysis of 5-year recurrence-free survival (**a**) and overall survival (**b**). UFT, tegafur-uracil; S-1, tegafur-gimeracil-oteracil; HR, hazard ratio; CI, confidence interval

The 5-year RFS rate was 56.1% in the UFT group and 59.6% in the S-1 group (Fig. 2a), and the 5-year OS rate was 78.3% in the UFT group and 73.1% in the S-1 group (Fig. 2b). The hazard ratio for recurrence (or all-cause mortality) and all-cause mortality in the S-1 group as compared with the UFT group was 1.14 (95% CI, 0.56 to 2.30; *p* value = 0.71) and 1.41 (95% CI, 0.53 to 3.73; *p* value = 0.48), respectively. The total number of recurrences was comparable between the UFT and S-1 groups (43.2% versus 38.8%; *p* = 0.68), and similarly, there was no statistical difference in sites of the first recurrence (UFT versus S-1), including local recurrence (10.8% versus 4.1%; *p* = 0.40), lymph nodes (16.2% versus 8.2%; *p* = 0.32), peritoneal carcinomatosis (16.2% versus 18.4%; *p* = 0.79), and hematogenous metastasis (16.2% versus 20.4%; *p* = 0.62), between these two groups (Supplementary Table 1). After Borrmann type, T, and N stages were adjusted, N stage (N3 versus N1) was the only significant predicting factor for RFS (HR = 2.86, 95% CI = 1.16 to 7.03; *p* = 0.02) while type of adjuvant chemotherapy (S-1 versus UFT) remained insignificant for RFS (HR = 1.25; 95% CI = 0.53 to 2.94; *p* = 0.61) (Table 2).

**Table 2:** Analysis of important risk factors on recurrence-free survival time using multivariate Cox proportional hazards model

	HR	95% CI	*p* value
Type of chemotherapy (S-1 vs UFT)	1.25	0.53-2.94	0.61
Borrmann type (III/IV vs I/II)	1.00	0.38-2.69	0.99
T stage (T3 vs T2)	2.99	0.96-9.36	0.05
T stage (T4 vs T2)	2.59	0.72-9.29	0.14
N stage (N2 vs N1)	1.25	0.53-2.92	0.61
N stage (N3 vs N1)	2.86	1.16-7.03	0.02*

*HR* hazard ratio, *CI* confidence interval, *UFT* tegafur-uracil, *S-1* tegafur-gimeracil-oteracil

*p value < 0.05

Twenty-seven (72.9%) patients in the UFT group and 36 (73.5%) patients in the S-1 group completed the adjuvant chemotherapy (*p* = 0.96), and for those completing the adjuvant chemotherapy, 10 (37.0%) patients in the UFT group, and 19 (52.8%) patients in the S-1 group required either dose reduction or schedule modification (*p* = 0.21) (Table 3). The most common reason for chemotherapy discontinuation was disease progression (4 patients in the UFT group versus 10 patients in the S-1 group), followed by adverse event (3 patients in the UFT group versus 3 patients in the S-1 group) (Table 3).

**Table 3:** Completion rates of adjuvant chemotherapy

	UFT	(*n* = 37)	S-1	(*n* = 49)	*p* value^#^
**Total number of completions**	**27**	**(72.9)**	**36**	**(73.5)**	**0.96**
Dose reduction or schedule modification	10	(37.0)	19	(52.8)	0.21
**Total number of discontinuations**	**10**	**(27.0)**	**13**	**(26.5)**	**0.96**
Adverse event	3	(30.0)	3	(23.1)	0.10
Disease progression	4	(40.0)	10	(76.9)	
Drop out	3	(30.0)	0	(0)	

Data are number of patients (%)

*UFT* tegafur-uracil, *S-1* tegafur-gimeracil-oteracil

^#^Comparison of completion rates between the UFT and S-1 groups

The most common all-grade adverse events were weight loss (27.0%) and constipation (27.0%), anemia (21.6%), followed by nausea (18.9%) in the UFT group, and were abdominal pain (34.7%), diarrhea (22.4%), followed by weight loss (20.4%) in the S-1 group. The most common ≥ grade 3 adverse event was nausea (5.4%) in the UFT group, and anemia (4.1%) and weight loss (4.1%) in the S-1 group. Hand-foot skin reaction was relatively rare and observed only in the mild form (< grade 3) in two (5.4%) patients in the UFT group and three (6.1%) patients in the S-1 group (Supplementary Table 2).

## Discussion

This study showed the comparable outcomes of 5-year RFS and OS for patients with pathological stage II-IIIB (excluding T1 cases; AJCC 8th) gastric cancer receiving adjuvant UFT or S-1 monotherapy following D2 gastrectomy by the same surgical team. The type of adjuvant chemotherapy, UFT or S-1, remained insignificant for RFS after Borrmann type, T, and N stages were adjusted. Although the adjuvant chemotherapy completion rate was similar between the UFT and S-1 groups, there were slightly more dose reductions and/or schedule modifications in the S-1 group.

Oral UFT has less been studied and reported as the adjuvant chemotherapy for advanced gastric cancer. Nakajima et al. reported an RCT regarding adjuvant treatment with UFT monotherapy for pathological stage T2N1-2 gastric cancer following gastrectomy in 2007 [9], showing that the 5-year RFS rate was 85% in the UFT and 68% in the surgery-only groups (*p* = 0.005), and the 5-year OS rate was 86% in the UFT and 73% in the surgery-only groups (*p* = 0.017), respectively. Although the results were promising, the treatment efficacy for pathological stage > T2N2 remained unclear. Later on, the ACTS-GC reported that adjuvant S-1 monotherapy had the 5-year RFS and OS survival benefits for pathological stage II-IIIB (T1 disease excluded; Japanese Classification of Gastric Carcinoma, 2nd English edition) gastric cancer in 2011. The 5-year RFS and OS rates in the ACTS-GC were greater in the S1 group (5-year RFS: 65.4%; 5-year OS: 71.7%) than in the surgery-only group (5-year RFS: 53.1%; 5-year OS: 61.1%) [7, 8]. Similarly, adjuvant capecitabine (Xeloda®), another oral fluoropyrimidine, plus oxaliplatin, was also shown effective for pathological stage II-IIIB (AJCC 6th) gastric cancer in the CLASSIC trial in 2012 [5, 6]. The 5-year RFS and OS rates in the CLASSIC trial were greater in the capecitabine-oxaliplatin group (5-year RFS: 68%; 5-year OS: 78%) than in the surgery-only group (5-year RFS: 53%; 5-year OS: 69%) [5, 6]. The successes of these two large RCTs established the role of oral fluoropyrimidines (S-1 and capecitabine) in the adjuvant setting for advanced gastric cancer; however, UFT seemed to be overlooked thereafter. In 2014, the SAMIT, a two-by-two factorial RCT, aimed initially to evaluate the survival benefit of sequential use of paclitaxel and oral fluoropyrimidines (UFT or S-1) for pathological T4a/4b gastric cancer [15]. The addition of paclitaxel was not associated with significant survival benefits; nevertheless, the results incidentally revealed adjuvant UFT monotherapy was inferior to S-1 monotherapy for patients with pathological T4a/4b gastric cancer following D2 gastrectomy (3-year RFS: 53% versus 58.2%; *p* < 0.001) [15].

The RFS and OS rates of the UFT or S-1 group in our study were inferior to those in the trial conducted by Nakajima et al. (T2N1-2), slightly inferior to those in the ACTS-GC (stage II-IIIB; Japanese classification) and CLASSIC (stage II-IIIB; AJCC 6th) trial, and superior to those in the SAMIT (T4a/b lesions) [5–9, 15]. The differences in survival outcomes between our study and aforementioned clinical trials may result from (1) various patient characteristics and inclusion criteria for adjuvant chemotherapy (including the effects of stage migration between different editions of gastric cancer classification); (2) different regimens, dosages, or durations of adjuvant chemotherapy; (2) retrospective nature of our study. Patients in the UFT/S1 group in this study comprised 56.8%/10.4% of pathological T4 lesions, 21.6%/14.3% of N3 (AJCC 8th) lesions (≥ 7 metastatic lymph nodes), and 70.3%/55.1% of stage III (AJCC 8th) gastric cancer. Patients randomized to the S-1 adjuvant chemotherapy arm in the ACTS-GC had 2.3% of pathological T4 lesions, 26.6% of ≥ 7 metastatic lymph nodes (equivalent to N3 in the AJCC 8th), and 48% of stage III (AJCC 6th) gastric cancer, and those in the CLASSIC trial had 1% of pathological T4 lesions, 31% of N2 (AJCC 6th; ≥ 7 metastatic lymph nodes) lesions (equivalent to N3 in the AJCC 8th), and 51% of stage III (AJCC 6th) gastric cancer [5, 7]. It was difficult to compare studies with different patient selection criteria. In this study, although the long-term oncological outcomes (5-year RFS and OS) were slightly inferior to those in the ACTS-GC and CLASSIC trial, there were actually more T4 lesions and stage III gastric cancers in our patients.

Comparisons between and the real-world experiences of the different oral fluoropyrimidines remain suboptimal. SAMIT, the only clinical trial evaluating the efficacy of UFT and S-1 for advanced gastric cancer, reported the 3-year RFS benefit for S-1 (HR = 0.81; *p* = 0.005); however, more extended oncological outcomes were still unknown [15]. In this study, the two adjuvant treatment cohorts were formed with the evolution of drug availability and reimbursement policy in the Taiwanese healthcare system, where UFT was available first then followed by S-1 10 years later, and the long-term oncological outcomes (5-year RFS and OS) were comparable between these two groups. Furthermore, the type of adjuvant chemotherapy (S-1 versus UFT) remained insignificant for RFS after Borrmann type, T, and N stages were adjusted. Compared to the SAMIT’s protocol, we adopted the same dosage of UFT but the duration was inevitably extended to 96 weeks. The longer duration (96 weeks versus 48 weeks in the SAMIT) and better chemotherapy complete rate (72.9% versus 60% in the SAMIT) of the UFT treatment may contribute to the survival benefit through the metronomic effect seen in this study [18, 19, 21–23]. Moreover, UFT was proved to have a unique antiangiogenic mechanism through its metabolites, γ-butyrolactone and γ-hydroxybutyric acid, in addition to direct cytotoxic effect from its active metabolite, 5-FU; nevertheless, whether these collateral metabolites could exert anti-tumor activity and provide survival benefit requires more investigations [24].

Tolerability and treatment completion rate also play an important role and affect long-term outcomes in adjuvant chemotherapy of advanced gastric cancer. Theoretically, S-1 should reduce gastrointestinal toxicity through the effect of oteracil, but any ≥ grade 3 gastrointestinal toxicities, including nausea, diarrhea, constipation, and abdominal pain, were similar between patients receiving UFT and S-1 treatments in this study (Supplementary Table 2) [13]. In line with our findings, the toxicity profiles of any ≥ grade 3 gastrointestinal toxicities were also similar between these two regimens in the SAMIT [15]. The optimal molar ratio of tegafur/gimeracil/oteracil and toxicity profile of S-1 were based on several animal studies but has less been compared with that of UFT [25, 26]. These discrepancies observed in the SAMIT and this study may also imply the necessity of re-evaluating the UFT in clinical use. Apart from the prolonged-course UFT treatment, the S-1 treatment was selectively extended to 12 courses (72 weeks) if dose reduction was required for adverse events. The rationale behind this strategy was also the concept of metronomic effect and to maintain the same total dosage of S-1 chemotherapy for dose-reduced S-1 group [21–23]. The strategy did not compromise the chemotherapy completion rates in this study, which were 72.9% in the UFT group and 73.5% in the S-1 group, compared to 65.8% in the ACTS-GC (S1 arm), 67% in the CLASSIC trial (capecitabine-oxaliplatin arm), and 60% in the SAMIT (UFT arm), and 62% in the SAMIT (S1 arm) [5, 7, 15]. Recently, emerging evidences also indicated that a longer duration of S-1 adjuvant chemotherapy had survival benefit for pathological stage II, III, and metastatic gastric cancer [27–29]; however, more prospective clinical trials are needed to confirm these results from retrospective data and further define a standard treatment duration with a corresponding dose.

Although this cohort study included limited number of patients, we presented the unique experiences and long-term oncological outcomes of using UFT or S-1 adjuvant monotherapy for advanced gastric cancer in Taiwan. The recent pandemic of COVID-19 reminds us that pharmaceutical supply chain may be unstable and re-evaluation of the pre-existing chemotherapeutic agents is necessary. Despite the fact that S-1 and capecitabine are the two main fluoropyrimidines used for advanced gastric cancer as adjuvant chemotherapy in this era, our study implies that UFT may also be a feasible alternative. Nevertheless, our study had some limitations. First, the patient numbers were much smaller than those in the RCTs, because we only included patients from the same surgical team to ensure the surgical quality, consistency of the D2 gastrectomy, and the same strategy for adjuvant chemotherapy. Second, the retrospective nature of the study and lack of additional active control groups using other adjuvant regimens, such as CAPOX (capecitabine + oxaliplatin) or P-HDFL (cisplatin + infusional high-dose 5-FU), also limited the comparisons and implications.

## Conclusions

Adjuvant UFT and S-1 monotherapy had comparable long-term outcomes for pathological stage II-IIIB gastric cancer following D2 gastrectomy based on the real-world data from our small cohort study.

## Supplementary Information

**Additional file 1: Supplementary Table 1.** Site of the first recurrence. **Supplementary Table 2.** Adverse events

## Data Availability

The datasets generated and/or analyzed during the current study are not publicly available but are available from the corresponding author on reasonable request.

## Abbreviations

RCT: Randomized controlled trial
DPD DIF: Dihydropyrimidine dehydrogenase inhibitory fluoropyrimidine
5-FU: 5-Flurouracil
UFT: Tegafur-uracil
S-1: Tegafur-gimeracil-oteracil
ACTS-GC: Adjuvant chemotherapy trial of TS-1 for gastric cancer
SAMIT: Stomach cancer Adjuvant Multi-Institutional group Trial
COVID-19: Coronavirus disease
AJCC: American Joint Committee on Cancer
RFS: Recurrence-free survival
OS: Overall survival
HR: Hazard ratio
CI: Confidence interval
