# Supplementary material for: Adjuvant tegafur-uracil (UFT) or S-1 monotherapy for advanced gastric cancer: a single center experience
Source: World J Surg Oncol. 2021 Apr 17;19:124. doi: 10.1186/s12957-021-02233-2 (PMC8053033; doi:10.1186/s12957-021-02233-2)
Supplement: Supplementary file 1 — Additional file 1: Supplementary Table 1. Site of the first recurrence. Supplementary Table 2. Adverse events [file 12957_2021_2233_MOESM1_ESM.docx]

**Supplementary Table 1.** Site of the first recurrence

|  | UFT (n=37) | S-1 (n=49) | *P* value |
| --- | --- | --- | --- |
| Total number of recurrences | 16 (43.2%) | 19 (38.8%) | 0.68 |
| Site |  |  |  |
| Local | 4 (10.8%) | 2 (4.1%) | 0.40 |
| Lymph nodes | 6 (16.2%) | 4 (8.2%) | 0.32 |
| Peritoneum | 6 (16.2%) | 9 (18.4%) | 0.79 |
| Hematogenous | 6 (16.2%) | 10 (20.4%) | 0.62 |

Data are number of patients (%).

UFT = tegafur-uracil; S-1 = tegafur-gimeracil-oteracil.

* *P* value < 0.05 after comparing between the UFT and S-1 groups.

**Supplementary Table 2.** Adverse events

|  | UFT (n=37) | | S-1 (n=49) | |
| --- | --- | --- | --- | --- |
|  | All grades | ≥ Grade 3 | All grades | ≥ Grade 3 |
| Leukopenia | 0 (0) | 0 (0) | 3 (6.1) | 0 (0) |
| Anemia | 8 (21.6) | 0 (0) | 9 (18.4) | 2 (4.1) |
| Thrombocytopenia | 0 (0) | 0 (0) | 2 (4.1) | 0 (0) |
| Elevated ALT | 0 (0) | 0 (0) | 2 (4.1) | 1 (2.0) |
| Elevated T-Bil | 0 (0) | 0 (0) | 3 (6.1) | 0 (0) |
| Elevated creatinine | 0 (0) | 0 (0) | 0 (0) | 0 (0) |
| Stomatitis | 1 (2.7) | 0 (0) | 4 (8.2) | 0 (0) |
| Weight loss | 10 (27.0) | 0 (0) | 10 (20.4) | 2 (4.1) |
| Nausea | 7 (18.9) | 2 (5.4) | 3 (6.1) | 1 (2.0) |
| Vomiting | 2 (5.4) | 0 (0) | 1 (2.0) | 0 (0) |
| Diarrhea | 2 (5.4) | 1 (2.7) | 11 (22.4) | 0 (0) |
| Constipation | 10 (27.0) | 0 (0) | 8 (16.3) | 0 (0) |
| HFSR | 2 (5.4) | 0 (0) | 3 (6.1) | 0 (0) |
| Skin rash | 1 (2.7) | 0 (0) | 6 (6.1) | 0 (0) |
| Abdominal pain | 3 (8.1) | 0 (0) | 17 (34.7) | 1 (2.0) |

Data are number of patients (%).

UFT = tegafur-uracil; S-1 = tegafur-gimeracil-oteracil; ALT = alanine aminotransferase; T-Bil = total bilirubin; HFSR = Hand-foot skin reaction.
